# P-1125. Impact of a Persuasive Antimicrobial Stewardship Program on Antibiotic Consumption and Mortality in a Neonatal Unit: A Before-and-After Study

**DOI:** 10.1093/ofid/ofae631.1312

**Published:** 2025-01-29

**Authors:** Juan Pablo Londono-Ruiz, Santiago Loaiza Betancourt, Diana Lucia Rodriguez-Acosta, Viviana Rodriguez

**Affiliations:** Hospital universitario Mayor Mederi, Bogota, Distrito Capital de Bogota, Colombia; Hospital Universitario Mayor - Mederi, Bogota, Distrito Capital de Bogota, Colombia; Hospital Universitario Mayor - Mederi, Bogota, Distrito Capital de Bogota, Colombia; Hospital Universitario Mayor - Mederi, Bogota, Distrito Capital de Bogota, Colombia

## Abstract

**Background:**

Antimicrobial Stewardship Programs (ASPs) have established themselves as the most effective strategy for reducing the consumption of unnecessary antibiotics, and thereby minimizing the emergence of antimicrobial resistance and adverse effects. However, implementing ASPs in neonatal units presents unique challenges due to their high use of antibiotics and the limited clinical tools available to diagnose infections. The aim of this study is to present the results of a before-and-after study that evaluated the impact of implementing an ASP with persuasive strategies on antibiotic consumption in a neonatal unit.

**Methods:**

The study was conducted in a neonatal unit of a general hospital that attends approximately 200-400 deliveries per month and has a high-risk obstetric program. The ASP was initiated with a weekly round of antibiotic control and an analysis and feedback strategy. Weekly education and training sessions were conducted for all staff, and the pediatric infectious diseases service was made available for permanent consultation. Local guidelines were developed and shared with all unit personnel, and the Days of Therapy (DOT) of the main antibiotics used was measured. Data analysis was performed using the R program.

**Figure:**
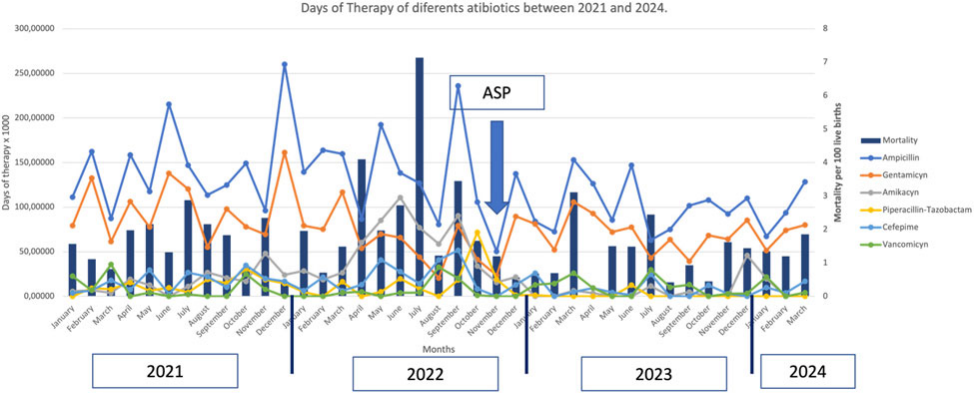

DOT and Mortality in Pre-ASP and Post-ASP periods

**Results:**

DOT measurements of ampicillin, gentamicin, amikacin, piperacillin-tazobactam, cefepime, and vancomycin were collected since 2021. In November 2022, a spot study was conducted to identify the microbiology of the neonatal unit, and the ASP was implemented. An analysis and feedback strategy were used, with additional support from a weekly round that included neonatologists, pediatricians, therapists, and nursing staff. The monthly DOT are shown in Figure 1. The average DDDs were compared between the period before the ASP and the period after, revealing a significant decrease in all antibiotics except for vancomycin (Table 1). A mortality rate of 2.08 per 100 live births was documented in the Pre-ASP period, which decreased to 1.2 per 100 live births in the post-ASP period.

**Figure:**
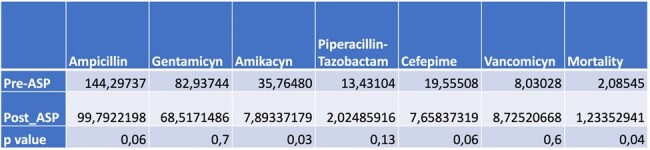

**Conclusion:**

Antibiotic stewardship programs that implement persuasive measures can be effective in reducing the consumption of most antibiotics in a neonatal unit. Mortality did not increase despite the reduced use of antibiotics.

**Disclosures:**

**All Authors**: No reported disclosures

